# Advertisements

**Published:** 1879-01-20

**Authors:** 


### *ADVERTISEMENTS.*

Our readers are requested to notice carefully the Advertisements. The advertising index on first cover page will enable you to find any one of them at a glance. There are a number of new advertisements in the present issue, to-wit:

C. Henri Leonard, Physician's Day Book; J. B. Lippincott; Ludden & Bates; Trommer Extract of Malt Company; McKesson & Robbins.

Others have changed their advertisements. Read them all, and ask your druggist and friends to patronize them.

